# Genetic variability and phylogenetic analysis of the VP2 gene of feline parvovirus in Harbin

**DOI:** 10.3389/fvets.2026.1783463

**Published:** 2026-02-25

**Authors:** Qingdong Luan, Chenxi Weng, Yiting Guo, Pengxin Yang, Guozhen Wu, Guojie Ding, Yijing Li

**Affiliations:** 1 College of Veterinary Medicine, Northeast Agricultural University, Harbin, China; 2 Harbin Vaccine Science Biotechnology Co., Ltd, Harbin, China

**Keywords:** feline parvovirus, genetic variation, Harbin, phylogenetic analysis, VP2 gene

## Abstract

**Introduction:**

In 2023, a total of 450 fecal samples were collected from healthy cats and those suspected of being infected with Feline Panleukopenia Virus (FPV) in Harbin, China.

**Methods:**

The FPV VP2 gene was detected using polymerase chain reaction (PCR). Positive samples were then subjected to VP2 sequence analysis, phylogenetic analysis, recombination analysis, and selective pressure analysis.

**Results:**

VP2 sequence analysis showed that the nucleotide similarity of the full-length VP2 gene ranged from 98.7 to 100%, with 19 amino acid mutations observed compared to the 2008 Felocell vaccine strain (GenBank: EU49868.1). Phylogenetic analysis demonstrated that all 42 detected FPV strains clustered with recent domestic isolates. Recombination analysis identified two strains (HRB2312 and HRB2324) as recombinants between FPV and Canine Parvovirus type 2c (CPV-2c). Selective pressure analysis did not detect any positively selected sites.

**Discussion:**

The findings suggest that the FPV lineages circulating in China have remained relatively stable in recent years, with evolution influenced by both random genetic drift and gene recombination. This study provides insights into the genetic diversity of FPV in Harbin, highlighting point mutations and recombination events. Further investigation is warranted to determine the antigenic compatibility of circulating recombinant strains with traditional vaccine strains.

## 1 Introduction

Feline parvovirus (FPV) is a non-enveloped, single-stranded DNA virus with a genome length of approximately 5.1 kb. Also referred to as the feline panleukopenia virus, it is classified as a variant of *Carnivore protoparvovirus* 1, genus *Protoparvoviru*s within the family *Parvoviridae* (1). Clinical symptoms following infection include high fever, vomiting, diarrhea, and severe leukopenia, as well as damage to the immune and nervous systems (2). Infected cats exhibit high morbidity and mortality rates, particularly in kittens under 3 months of age, who are especially susceptible to the virus. Infected cats primarily shed the virus through feces, vomit, and ocular secretions (2–4). Due to FPV’s strong resistance to physical and chemical factors, it can remain infectious in the environment for several months or even years (5–8).

Canine parvovirus (CPV) and feline parvovirus (FPV) are closely related in pathogen evolution. FPV was first identified in 1928 and subsequently isolated via tissue culture in 1964 (9, 10). CPV-2 was discovered in 1978 and is believed to have originated from FPV or other closely related parvovirus (11). FPV and CPV-2 share identical genomic structures, comprising two open reading frames (ORFs): ORF1 and ORF2. ORF1 encodes two non-structural proteins, NS1 and NS2 (12, 13); ORF2 encodes the structural proteins VP1 and VP2 through alternative splicing of the same mRNA. VP2 is located at the C-terminal end of VP1 and constitutes 88–90% of the structural protein (14, 15). The VP2 protein is the most important structural component and a key target for generating neutralizing antibodies, determining host range, tissue tropism, and hemagglutination (16–20). Alterations in VP2 amino acids can lead to viral evolution and the emergence of different antigenic variants (16, 17). FPV and CPV-2 can be distinguished by specific mutations in the VP2 protein, including L80R, L90N, V103A, V232I, E323N, N564S, and A568S (16–20). Therefore, genetic evolutionary analysis of the VP2 gene can effectively distinguish between different FPV strains, revealing the virus’s evolutionary patterns and transmission trends.

Currently, prevention of FPV relies primarily on vaccination. However, frequent reports of clinical vaccine failure have raised concerns about the efficacy of vaccine protection (21–23). In addition to host factors such as maternal antibody interference and individual immune status (24, 25), the continuous evolution of the virus is also an important factor that cannot be overlooked. FPV evolution is mainly driven by random genetic drift (26) and genetic recombination (13). In recent years, the FPV VP2 gene has exhibited a sustained evolutionary trend. Surveillance data from southwestern China indicate that the positivity rate for the FPV VP2 A91S variant has risen annually from 2019 to 2021, becoming the dominant circulating strain (27). The A91S mutant strain exacerbates lungs infections (28). Additionally, FPV variants derived from giant pandas have been found to infect human cells *in vitro*, suggesting a potential zoonotic transmission risk (19). However, there is currently a lack of systematic research on the prevalence, genetic characteristics, and evolutionary dynamics of FPV in Harbin. Therefore, this study collected fecal samples from healthy and suspected Parvovirus-infected cats in Harbin in 2023, performed PCR amplification of the full-length VP2 gene, and conducted VP2 sequence analysis, phylogenetic analysis, recombination analysis, and selection pressure analysis. The aim of this study to clarify the prevalence, molecular characteristics, and evolutionary patterns of FPV in Harbin, providing scientific evidence for vaccine evaluation and optimization of control strategies.

## 2 Materials and methods

### 2.1 Sample collection

A total of 450 fecal samples were collected in 2023 from healthy cats and those suspected of being infected with parvovirus at veterinary clinics in Harbin (Table 1). The samples documented the age, clinical symptoms, gender and vaccination status of the cats. The age of the cats enrolled ranged from 2 months to 8 years. Among the total 450 cats, the male-to-female ratio was approximately 1:1.2. Recorded clinical symptoms included vomiting, diarrhea, hematochezia, and fever. After collection, the samples were placed in 0.1 M PBS (pH = 7.4) containing 100 U/mL penicillin and 100 U/mL streptomycin, and stored at −80 °C.

**Table 1:** Clinical signs in cats testing positive for FPV in this study.

Name	GenBank	Age (mon)	Gender	Clinical sign	Vaccine history
HRB2301	PV948245	6	Male	Vomiting, fever, diarrhea	−
HRB2302	PV948246	5	Male	Fever, hematochezia, vomiting	−
HRB2303	PV948243	12	Female	Fever, diarrhea	+−
HRB2304	PV948244	10	Female	Vomiting, diarrhea	+
HRB2305	PV948247	24	Female	No clinical sign	+
HRB2306	PV948248	8	Male	Fever, diarrhea	−
HRB2307	PV948249	4	Female	Diarrhea, fever	−
HRB2308	PV948250	4	Female	Diarrhea, fever	−
HRB2309	PV948251	7	Male	Diarrhea, fever	−
HRB2310	PV948252	2	Male	Fever, diarrhea, vomiting	−
HRB2311	PV948211	7	Female	Fever, diarrhea	−
HRB2312	PV948212	2	Male	Diarrhea, fever, hematochezia	−
HRB2313	PV948213	11	Female	Fever, vomiting	−
HRB2314	PV948214	7	Male	Fever, diarrhea	−
HRB2315	PV948215	5	Female	Fever, vomiting, diarrhea	−
HRB2316	PV948216	9	Male	Fever, vomiting, diarrhea	−
HRB2317	PV948217	14	Female	Diarrhea	+
HRB2318	PV948218	13	Female	Diarrhea, vomiting	+−
HRB2319	PV948219	10	Female	Fever, diarrhea	−
HRB2320	PV948220	5	Female	Diarrhea, fever, hematochezia	−
HRB2321	PV948221	4	Male	Diarrhea, fever, hematochezia	−
HRB2322	PV948222	5	Male	Diarrhea, fever, hematochezia	−
HRB2323	PV948223	8	Female	Fever, vomiting, diarrhea	−
HRB2324	PV948224	9	Female	Fever, vomiting, diarrhea	−
HRB2325	PV948225	3	Male	Diarrhea, fever, hematochezia	−
HRB2326	PV948226	7	Female	Fever, vomiting, diarrhea	−
HRB2327	PV948227	8	Male	Fever, vomiting, diarrhea	−
HRB2328	PV948228	5	Male	Fever, hematochezia	−
HRB2329	PV948229	5	Female	Fever, hematochezia	−
HRB2330	PV948230	4	Male	Diarrhea, fever, hematochezia	−
HRB2331	PV948231	4	Male	Diarrhea, fever, hematochezia	−
HRB2332	PV948232	6	Female	Fever, hematochezia	−
HRB2333	PV948233	8	Male	Fever, vomiting, diarrhea	−
HRB2334	PV948234	9	Female	Fever, vomiting, diarrhea	−
HRB2335	PV948235	8	Female	Fever, vomiting, diarrhea	−
HRB2336	PV948236	4	Male	Diarrhea, fever, hematochezia	−
HRB2337	PV948237	15	Female	Diarrhea	+
HRB2338	PV948238	2	Male	Diarrhea, fever, hematochezia	−
HRB2339	PV948239	2	Male	Diarrhea, fever, hematochezia	−
HRB2340	PV948240	4	Male	Diarrhea, fever, hematochezia	−
HRB2341	PV948241	11	Female	Fever, vomiting, diarrhea	+−
HRB2342	PV948242	3	Male	Diarrhea, fever, hematochezia	−

+, Fully vaccinated; −, Not vaccinated; +−, Incomplete vaccination.

### 2.2 DNA extraction and amplification of VP2

Total DNA was extracted from samples using the DNAiso kit (Vazyme Biotech Co., Ltd., Nanjing, China) and used in the PCR assay for FPV detection according to the method described by Ye et al. (29) the forward primer was 5′- CTTTGCCTCAATCTGAAGGAG-3′, and the reverse primer was 5′-GAATTGGATTCCAAGTATGAG-3′ Reactions were conducted in a 20 μL system comprising 10 μL of master mix (Vazyme Biotech Co., Ltd., Nanjing, China), 0.5 μL each of forward and reverse primers, 1 μL of template, and ddH_2_O to a final volume of 20 μL. The detection protocol included a 3-min pre-heating step at 95 °C, followed by 35 cycles of 15 s at 95 °C, 15 s at 55 °C, and 15 s of extension at 72 °C, with a final extension of 5 min at 72 °C. The products were analyzed by 1% agarose gel.

Positive samples underwent amplification of the full-length VP2 gene using the previously reported method (30). The amplified fragment of the target gene was 1,776 bp. The amplification products were recovered and purified according to the kit instructions (Vazyme Biotech Co., Ltd., Nanjing, China). The purified target fragments were ligated into the pMD19-T vector (Takara Biotechnology Co., Ltd., Beijing, China) and transformed into DH5α *E. coli* (Weidi Biotech Co., Ltd., Shanghai, China). Positive colonies were selected for identification, and the positively identified clones were sequenced by Sangon Biotech (Shanghai).

### 2.3 Phylogenetic analysis

A total of 40 reference sequences of FPV, MEV (Mink Enteritis Virus), and CPV-2 were retrieved from GenBank. The nucleotide and amino acid sequences of the VP2 gene were aligned using DNASTAR software. A phylogenetic tree was constructed using the Maximum Likelihood method based on the Tamura-Nei model in MEGA version 7.0. Branch support was assessed with 1,000 bootstrap replicates.

### 2.4 Recombination and selection pressure analysis

To detect whether a recombination event occurred in the VP2 gene, recombination analysis was performed using RDP4^1^ and SimPlot^2^ based on the sequences used in the phylogenetic analysis. RDP4 integrates seven algorithms—Bootscan (31), Chimera (32), GeneConv (33), MaxChi (34), RDP (35), SiScan (36), and 3Seq (37)—to detect recombination events and identify potential recombination breakpoints. The highest acceptable *p*-value was set at 0.05. The recombination events detected by RDP4 were further confirmed using SimPlot software.

The SLAC and MEME programs in the online software Datamonkey^3^ were utilized to analyze whether the FPV VP2 protein is under positive selection pressure and to identify potential positive selection sites. The SLAC program calculates the ratio of nonsynonymous substitutions (dN) to synonymous substitutions (dS), represented by the *ω* value. When *ω* > 1, this indicates positive selection. The MEME program calculates the *p*-value (significance of positive selection), with a *p*-value < 0.05 considered evidence of positive selection. To minimize the risk of false positives, both methods were employed in combination. A site was deemed to be under positive selection when *ω* (dN/dS) > 1 and the *p*-value < 0.05.

## 3 Result

### 3.1 Sample collection and VP2 amplification

A total of 450 samples were collected, of which 42 tested positive, yielding a positivity rate of 9.3% (Table 1). All 42 positive samples successfully amplified the full VP2 gene, with GenBank accession numbers assigned as PV948211-PV948252. Detailed information on the 42 cats is presented in Table 1. Except for one case, other FPV-positive samples, clinical manifestations included fever in 88.1% of cases, vomiting in 38.1%, diarrhea in 85.7% and hematochezia in 38.1%. In terms of vaccination status, 2 samples were incomplete vaccination, 4 were vaccinated, and 36 had not received vaccination. The age of the FPV-positive cats ranged from 2 to 24 months. Analysis of the VP2 key amino acid sites revealed that all 42 samples were identified as FPV, with no detection of CPV-2a, CPV-2b, or CPV-2c. The primers used in this study are listed in Table 2. Analysis of the nucleotide similarity among the 42 FPV VP2 sequences showed a high degree of similarity, ranging from 98.7 to 100%.

**Table 2:** Primers used in this study.

Primer	Sequence 5′ to 3′	Length(bp)	Reference
FPV-F1	CTT TGC CTC AAT CTG AAG GAG	768	28
FPV-R1	GAA TTG GAT TCC AAG TAT GAG		
FPV-VP2F2	AGAGACAATCTTGCACCAAT	1776	29
FPV-VP2R2	ATGTTAATATAATTTTCTAGGTGCT		

### 3.2 VP2 gene sequence analysis

Analysis of the VP2 amino acid sequences from 42 FPV strains revealed that, compared to the reference sequence of the FPV strain Felocell (vaccine strain, GenBank: EU49868.1) from 2008, a total of 39 strains (92.9%) exhibited mutations at positions 6, 48, 50, 75, 76, 79, 80, 91, 93, 103, 122, 163, 222, 232, 331, 433, 562, 564, and 568, with varying frequencies and types of amino acid substitutions. Among these, the A91S, I232L, and L562V mutations were present, representing a 100% mutation rate. Notably, mutations at positions 6, 48, 50, 75, 76, 79, 122, 163, 222, 331, and 433 were novel and had not been reported in previous studies. Interestingly, HRB2331 shared identical amino acids with CPV-2, CPV-2a, CPV-2b, and CPV-2c at positions 80, 93, and 103; similarly, HRB2312 and HRB2324 exhibited the same amino acids as these CPV variants at positions 562, 564, and 568 (Table 3).

**Table 3:** Amino acid mutations in the VP2 proteins of FPVs and CPVs.

Accession number	Amino acid^a^																		
	6	48	50	75	76	79	80	91	93	103	122	163	222	232	331	433	562	564	568
EU498681.1	V	Q	E	E	S	Y	K	A	K	V	N	K	H	I	M	T	L	N	A
EU914139	V	Q	E	E	S	Y	R	A	N	A	N	K	H	I	M	T	V	S	G
FJ005233	V	Q	E	E	S	Y	R	A	N	A	N	K	H	I	M	T	V	S	G
M24003	V	Q	E	E	S	Y	R	A	N	A	N	K	H	I	M	T	V	S	G
M74849	V	Q	E	E	S	Y	R	A	N	A	N	K	H	I	M	T	V	S	G
PV948245	−	−	−	−	−	−	−	S	−	−	−	−	−	V	−	−	V	−	−
PV948246	−	−	−	−	−	−	−	S	−	−	−	−	−	V	−	A	V	−	−
PV948243	−	−	−	−	−	−	−	S	−	−	−	−	−	V	−	−	V	−	−
PV948244	−	−	−	−	−	−	−	S	−	−	−	−	−	V	−	−	V	−	−
PV948247	−	R	−	Q	−	−	−	S	−	−	−	−	−	V	−	−	V	−	−
PV948248	−	−	−	−	−	−	−	S	−	−	−	−	−	V	−	−	V	−	−
PV948249	−	−	−	−	−	−	−	S	−	−	−	−	−	V	−	−	V	−	−
PV948250	−	−	−	−	−	C	−	S	−	−	S	−	−	V	−	−	V	−	−
PV948251	−	−	−	−	−	C	−	S	−	−	S	−	−	V	−	−	V	−	−
PV948252	−	−	−	−	−	C	−	S	−	−	S	−	−	V	−	−	V	−	−
PV948211	−	−	−	−	−	C	−	S	−	−	S	−	−	V	−	−	V	−	−
PV948212	−	−	−	−	−	−	−	S	−	−	−	N	−	V	T	−	V	S	G
PV948213	−	−	−	−	−	C	−	S	−	−	S	−	−	V	−	−	V	−	−
PV948214	−	−	−	−	−	−	−	S	−	−	−	−	−	V	−	−	V	−	−
PV948215	−	−	−	−	−	−	−	S	−	−	−	−	−	V	−	−	V	−	−
PV948216	−	−	−	−	−	−	−	S	−	−	−	−	−	V	−	−	V	−	−
PV948217	−	−	−	−	−	−	−	S	−	−	−	−	−	V	−	−	V	−	−
PV948218	−	−	−	−	−	−	−	S	−	−	−	−	−	V	−	−	V	−	−
PV948219	−	−	−	−	−	−	−	S	−	−	−	−	−	V	−	−	V	−	−
PV948220	−	−	−	−	−	−	−	S	−	−	−	−	−	V	−	−	V	−	−
PV948221	−	−	−	−	−	−	−	S	−	−	−	−	−	V	−	−	V	−	−
PV948222	−	−	−	−	−	C	−	S	−	−	S	−	−	V	−	−	V	−	−
PV948223	−	−	−	−	−	C	−	S	−	−	S	−	−	V	−	−	V	−	−
PV948224	−	−	−	−	−	−	−	S	−	−	−	−	−	V	−	−	V	−	−
PV948225	−	−	−	−	−	−	−	S	−	−	−	−	−	V	−	−	V	−	−
PV948226	−	−	A	−	T	−	−	S	−	−	−	−	−	V	−	−	V	−	−
PV948227	−	−	−	−	−	−	R	S	N	A	−	−	−	V	−	−	V	−	−
PV948228	−	−	−	−	−	−	−	S	−	−	−	−	−	V	−	−	V	−	−
PV948229	−	−	A	−	T	−	−	S	−	−	−	−	−	V	−	−	V	−	−
PV948230	−	−	−	−	−	−	−	S	−	−	−	−	R	V	−	−	V	−	−
PV948231	−	−	−	−	−	−	R	S	N	A	−	−	−	V	−	−	V	−	−
PV948232	−	−	−	−	−	−	−	S	−	−	−	−	−	V	−	−	V	−	−
PV948233	−	−	−	−	−	−	−	S	−	−	−	−	−	V	−	−	V	−	−
PV948234	−	−	−	−	−	−	−	S	−	−	−	−	−	V	−	−	V	−	−
PV948235	−	−	−	−	−	C	−	S	−	−	S	−	−	V	−	−	V	−	−
PV948236	−	−	−	−	−	C	−	S	−	−	S	−	−	V	−	−	V	−	−
PV948237	−	−	−	−	−	C	−	S	−	−	S	−	−	V	−	−	V	−	−
PV948238	−	−	−	−	−	C	−	S	−	−	S	−	−	V	−	−	V	−	−
PV948239	−	−	−	−	−	−	−	S	−	−	−	−	−	V	−	−	V	−	−
PV948240	−	−	−	−	−	−	−	S	−	−	−	−	−	V	−	−	V	−	−
PV948241	−	−	−	−	−	−	−	S	−	−	−	−	−	V	−	−	V	−	−
PV948242	−	−	−	−	−	−	−	S	−	−	−	−	−	V	−	−	V	−	−

^a^Amino acid positions identical to the reference sequence are indicated by (−); A, Ala; E, Glu; G, Gly; K, Lys; L, Leu; M, Met; N, Asn; R, Arg; S, Ser; V, Val; Q, Gln; H, His; I, Ile; T, Thr; C, Cys.

### 3.3 Phylogenetic analysis

**Figure 1:**
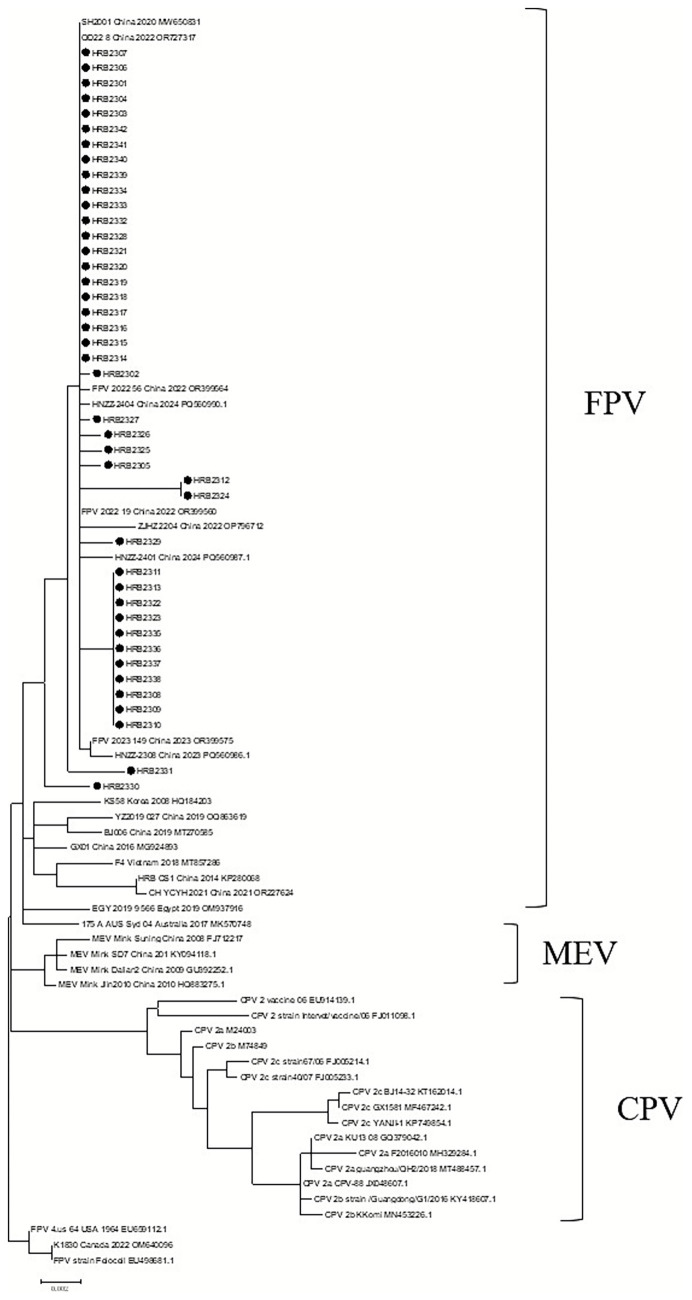
Maximum-likelihood tree constructed based on the full-length VP2 genes of FPV, CPV, and MEV. The tree was built using full-length VP2 gene sequences from 82 strains of FPV, CPV, and MEV, with 1,000 bootstrap replicates performed using MEGA 7 software. The FPV strain Felocell was used as the outgroup. Sequences are annotated with virus type (FPV/CPV/MEV), country and year of isolation, and GenBank accession number. Sequences obtained in this study are marked with black dots.

A maximum likelihood (ML) phylogenetic tree was constructed based on VP2 gene sequences from 40 reference strains of FPV, CPV, and MEV collected from various countries. The resulting tree was clearly divided into three distinct clades: the FPV clade, the MEV clade, and the CPV clade (Figure 1).

The FPV branch primarily consists of two sub-branches. The first sub-branch includes nine strains isolated domestically after 2020. The second sub-branch mainly comprises strains isolated abroad, including those from South Korea, Vietnam, Egypt, and Australia, as well as strains isolated domestically prior to 2020. Only one strain, YCYH, represents a strain isolated in China in 2021.

All 42 FPV strains analyzed in this study clustered within the first subclade. Within this subclade, three distinct clusters with genetic distances of less than 0.001 were identified. The first cluster was characterized by the presence of mutations A91S, I232V, and L562V. The second cluster exhibited mutations Y79C, N122S, A91S, I232V, and L562V. The third cluster, composed of strains HRB2312 and HRB2324, contained not only A91S, I232V, and L562V, but also additional mutations, including K163N, M331T, N564S, and A568G.

### 3.4 Recombination and selection pressure analyses

**Figure 2:**
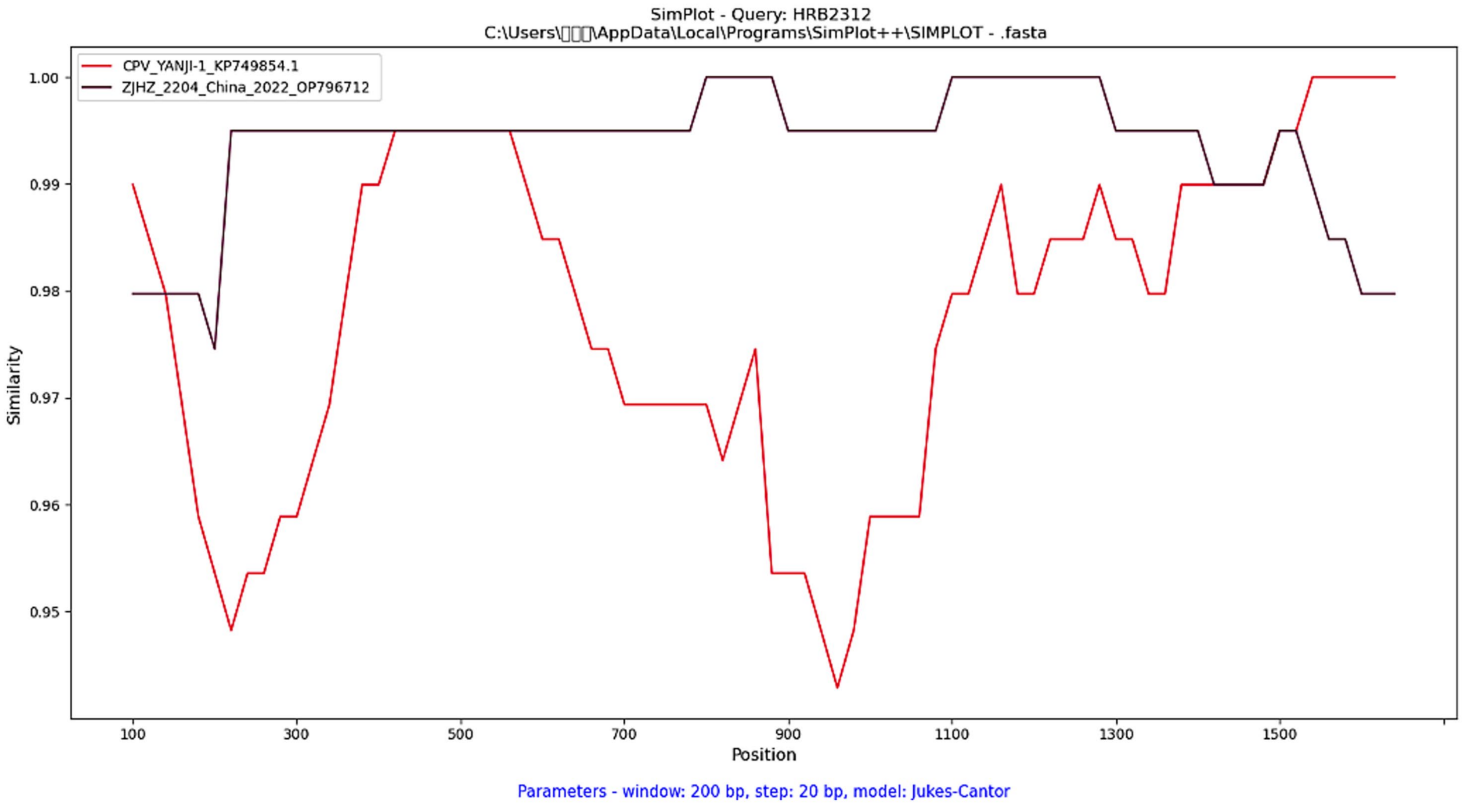
Recombination analysis of the recombinant strains was conducted using SimPlot software. Recombination breakpoints are indicated by the intersections of the red and brown lines, respectively, within a 200 bp-wide window with a 20 bp step size.

Recombination analysis of the 42 FPV VP2 sequences in this study was conducted using RDP4 software. The results indicated that HRB2312 and HRB2324 were identified as recombinant strains between FPV and CPV by GeneConv, Bootscan, and 3Seq, with *p*-values of 2.643 × 10^−2^, 3.005 × 10^−3^, and 5.005 × 10^−5^, respectively, all below the significance threshold of 0.05. The recombination breakpoints were identified at nucleotide positions 125 and 1,472. The region from 125 to 1,472 was closely related to the FPV strain ZJHZ-2204 (GenBank: OP796712), while the regions from 1 to 124 and from 1,473 to 1,755 showed close genetic relatedness to the CPV-2c strain YANJI-1 (GenBank: KP749854.1). Validation of the recombination event using SimPlot software confirmed the findings (Figure 2). Both RDP4 and SimPlot consistently identified HRB2312 and HRB2324 as recombinant strains derived from FPV and CPV-2c.

Selection pressure analysis was conducted using the SLAC and MEME algorithms available on the Datamonkey online platform. In evolutionary terms, positively selected sites indicate advantageous mutations, negatively selected sites represent deleterious mutations, and neutral sites refer to mutations with no significant effect on fitness. Results from the SLAC model showed that the dN/dS (*ω*) values at all codon positions were less than 1, indicating the absence of positively selected sites; all observed mutations were either neutral or under purifying (negative)selection. The MEME model detected three sites—35 (*p* = 0.031), 270 (*p* = 0.004), and 324 (*p* = 0.021)—with statistically significant evidence (*p* < 0.05) of positive selection. However, to minimize the risk of false positives, only sites identified by both SLAC and MEME as positively selected were considered reliable. Therefore, it is concluded that no positively selected sites were definitively identified in the VP2 gene of the FPV strains analyzed in this study.

## 4 Discussion

This study systematically investigated the prevalence, genetic characteristics, and evolutionary dynamics of FPV in Harbin based on the full-length VP2 gene sequences. It revealed unique amino acid variation patterns in locally circulating strains and identified recombinant strains of FPV and CPV-2c based on the VP2 gene. These findings indicate that FPV in Harbin is undergoing active evolutionary processes, with genetic diversity driven by random genetic drift and genetic recombination. This phenomenon holds significant implications for evaluating the efficacy of existing vaccines and formulating regional control strategies.

Random genetic drift is one mechanism driving the evolution of FPV. Analysis of amino acid sites in the VP2 protein revealed numerous amino acid mutations between the sequence in this study and the reference sequence of the FPV Felocell Vaccine (GenBank: EU49868.1). Among these mutations, A91S, I232V, and L562V have been reported in previous studies (18, 38, 39) In this investigation, the mutation rates for A91S, I232V, and L562V reached 100%, suggesting that these variations may have become essential genetic background for the current FPV. The VP2 contains two antigenic sites, A and B. Antigenic site Ais composed of amino acids at loop 1, loop 2, loop 4, and positions 93, 222, 224, and 426, while antigenic site B is formed by loop 3 and amino acids at positions 299, 300, and 302 (40, 41). Literature data indicate that the mutation at position 93 of the amino acid affects TfR binding, host range, and species-specific antibody responses in dogs and cats (40, 42–46). In this study, mutations at positions 222 (loop 2) and 433 (loop 4) were identified as part of antigenic site A of FPV VP2, which includes position 93. These mutations may alter the binding affinity of VP2 to the transferrin receptor (TfR) or the spatial conformation of neutralizing epitopes, potentially changing the virus’s cell tropism and antigenicity.

FPV not only drives evolution through random genetic drift but also undergo recombination by exchanging genetic material with CPV (47, 48). The discovery of the FPV-CPV-2c recombinant strains provides crucial evidence for understanding how genetic recombination drives viral evolution. Based on the full-length VP2gene sequence analysis, HRB2312 and HRB2324 were identified as recombinant strains. These recombinant strains exhibit a unique genomic structure: nucleotides 125–1,472 are similar to those of FPV isolates, while nucleotides 1–124 and 1,473–1,755 are closely related to CPV-2c isolates. Previously considered highly conserved loci, including positions 80, 93, 103, 564, and 568, which were used to differentiate FPV and CPV, showed typical CPV-like amino acid residues at positions564 and 568 (14, 39). Studies have shown that amino acid residues at positions 564 and 568 are critical for efficient viral replication (49, 50). Crystallographic analysis indicates that these residues are located in the shoulder region of the VP2 threefold spike. The amino acid composition of this region is essential for maintaining capsid stability and ensuring efficient viral particle assembly (41, 51). This suggests that the recombination event was not a neutral genetic exchange but rather a functionally driven evolutionary event. It allowed the virus to specifically acquire CPV-2c fragments, which may optimize capsid stability, assembly efficiency, and tissue tropism, while retaining the fundamental replication framework of FPV. Meanwhile, minor alterations in this region are associated with viral tissue tropism *in vitro* and pathogenicity *in vivo* (41, 51), indicating that the recombination event may directly give rise to viral variants with novel phenotypic traits. Additionally, the parentage information of the recombinant strains indicates that the primary parent is the FPV isolated ZJHZ-2204 (OP796712) from Zhejiang in 2022, and the secondary parent is the CPV-2c isolated YANJI-1 (KP749854.1) from Yanji in 2014. The temporal and geographical span suggests the possibility of unknown co-circulation events between FPV and CPV-2c. During co-circulation, FPV was found to have incorporated antigenic fragments of CPV-2c. The resulting recombinant strains might exhibit an expanded host range and distinct antigenicity compared to the original parental strains, thus avoiding the risk of virus extinction due to pressure from a single host or vaccines.

The discovery of point mutations in the FPV VP2 gene and the emergence of recombinant strains present new challenges to current prevention strategies that primarily rely on vaccination against FPV. The degree of antigenic match between vaccine strains and circulating field strains is a key determinant of cross-protective efficacy. In the case of CPV, have confirmed significant antigenic differences between variant strains and the original strains by Cavalli et al. (52). Therefore, it is essential to establish a long-term epidemiological surveillance system for FPV and conduct serological cross-neutralization assays based on both circulating and vaccine strains. Such efforts will help evaluate the protective efficacy of existing vaccines against current field strains, promote the iterative optimization of vaccines, and ensure that vaccination strategies remain effective against the virus’s adaptive evolution.

## Data Availability

The datasets presented in this study can be found in online repositories. The names of the repository/repositories and accession number(s) can be found below: https://www.ncbi.nlm.nih.gov/genbank/, PV948245, PV948246, PV948243, PV948244, PV948247, PV948248, PV948249, PV948250, PV948251, PV948252, PV948211, PV948212, PV948213, PV948214, PV948215, PV948216, PV948217, PV948218, PV948219, PV948220, PV948221, PV948222, PV948223, PV948224, PV948225, PV948226, PV948227, PV948228, PV948229, PV948230, PV948231, PV948232, PV948233, PV948234, PV948235, PV948236, PV948237, PV948238, PV948239, PV948240, PV948241, PV948242.

## Footnotes

1
http://web.cbio.uct.ac.za/~darren/rdp.html

2
https://github.com/Stephane-S/Simplot_PlusPlus

3
https://www.datamonkey.org/
